# Recent Advances in the Roles of MicroRNA and MicroRNA-Based Diagnosis in Neurodegenerative Diseases

**DOI:** 10.3390/bios12121074

**Published:** 2022-11-24

**Authors:** Juan Zhang, Zhu Chen, Hui Chen, Yan Deng, Song Li, Lian Jin

**Affiliations:** Hunan Key Laboratory of Biomedical Nanomaterials and Devices, Hunan University of Technology, Zhuzhou 412007, China

**Keywords:** neurodegenerative diseases, microRNA, early diagnosis, Alzheimer’s disease, Parkinson’s disease

## Abstract

Neurodegenerative diseases manifest as progressive loss of neuronal structures and their myelin sheaths and lead to substantial morbidity and mortality, especially in the elderly. Despite extensive research, there are few effective treatment options for the diseases. MicroRNAs have been shown to be involved in the developmental processes of the central nervous system. Mounting evidence suggest they play an important role in the development of neurodegenerative diseases such as Alzheimer’s disease and Parkinson’s disease. However, there are few reviews regarding the roles of miRNAs in neurodegenerative diseases. This review summarizes the recent developments in the roles of microRNAs in neurodegenerative diseases and presents the application of microRNA-based methods in the early diagnosis of these diseases.

## 1. Introduction

Neurodegenerative diseases are the degeneration of the central nervous system (CNS), which is characterized by the chronic progressive loss of neurons and their myelin sheath, resulting in structural and functional damage [1]. The development of these diseases, such as Alzheimer’s disease (AD), Parkinson’s disease (PD), Amyotrophic lateral sclerosis (ALS) and Huntington’s disease (HD), are believed to be closely related to age [2]. With the improvement of the quality of life, people’s life expectancy will also increase significantly, which will aggravate the prevalence of these diseases [3,4]. About 50 million people currently suffer from AD, which is expected to grow to 130 million by 2050 [5]. Thus, many efforts have been made in exploring the pathological mechanisms of these diseases to improve the situation. AD, as one of the most prevalent neurodegenerative diseases, has been reported to be associated with overproduction of amyloid beta protein (Aβ) and hyperphosphorylation of tau protein [6]. As the second most common neurodegenerative disease, PD is believed to result from the progressive loss of dopaminergic neurons in the substantia nigra with a decrease in dopamine levels in the striatum [7]. ALS is a type of neurodegenerative disease characterized by the death of motor neurons in the motor cortex, spinal cord, and spinal cord interneurons [8]. The main pathological changes in HD are striatal and cortical degeneration [9]. However, the pathological mechanisms of neurodegenerative diseases have not been elucidated, which results in limited diagnosis and therapies for these diseases. Considering the majority of neurodegenerative diseases cause irreversible damage to the CNS as the diseases develop, the early diagnosis of these diseases is of great significance for improving the current state. Non-invasive and point-of-care platforms that provide early diagnosis, real-time response, and low detection limits are also important for disease monitoring and clinical applications [10,11,12,13,14]. There are various methods for early diagnosis of diseases, such as biosensing, aptamers, nanomaterials, etc. [14,15,16,17,18,19,20,21]. The main focus here is on miRNAs. Studies have shown that it was possible to use magnetic bead chemiluminescence for ultrasensitive detection of miRNA in gastric cancer plasma [22], which indicates a breakthrough in the clinical application of miRNA if it could be used in neurodegenerative diseases, compared with some other traditional methods [23,24,25,26,27,28,29], which not only allows rapid detection of disease but also saves time and effort [30,31,32,33,34]. Therefore, miRNA detecting is of great importance [35,36,37].

**Figure 1:**
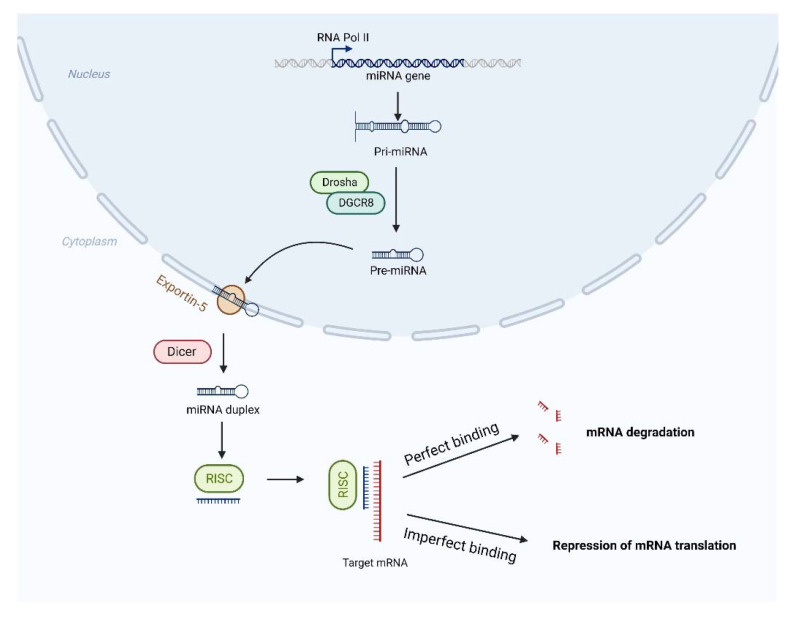
Schematic representation of the biosynthesis process of miRNA. Genes encoding miRNAs are transcribed to pri-miRNA, which is then converted to pre-miRNA. Pre-miRNA is transported to the cytoplasm and converted into miRNA duplexes which, together with the protein, creates an effector complex, RNA-induced silencing complex. Meanwhile, miRNA duplexes are converted into single strands, one of which remains as mature miRNA. At last, mature miRNA does its role in mRNA translation through binding to their target regions [48].

MicroRNAs (miRNAs) are non-coding single-stranded small RNAs with short sequences of ~22 nucleotides that regulate post-transcriptional gene expression by directing target mRNA cleavage or translational inhibition. It has been reported that miRNAs play critical roles in regulating a wide variety of biological processes, such as morphogenesis, cell fate, synaptic plasticity, apoptosis, mRNA splicing, DNA methylation, circadian rhythms, endocrinological regulation, angiogenesis, immunomodulation, and neuroprotection [38,39]. MiRNAs play the regulatory role mainly by pairing with the 3- or 5-terminal untranslated region bases of mRNA to inhibit gene expression at the post-transcriptional level [40] (Figure 1). Hundreds of miRNAs are known to play important roles in maintaining the biological functions in CNS [41,42]. During normal brain development, the expression of approximately 20% of miRNAs changes significantly, among which miR-124, miR-155, and miR-689 are closely related to the microglial activation phenotype in the CNS [43]. At present, more and more miRNAs have been found to be involved in the pathogenesis, progression, diagnosis and treatment of CNS diseases, including AD, PD, HD, and others [44]. Dysregulation of miRNAs may promote neurodegeneration and ultimately lead to neurodegenerative diseases. MiR-223-3p has been implicated in regulating a wide range of cellular processes and many types of pathological processes, such as cancer and autoimmunity [45]. Studies have shown that, compared with the normal group, the concentration of miR-223-3p is decreased in AD and increased in PD [45]. Hoss et al. [46] found high levels of miR-10b-5p and miR-486-5p in the brain and blood samples of HD patients and also showed that miR-10b-5p is closely related to disease stage, age of onset, and neuropathology in the brain. In ALS, miR-128-3p and miR-103b-3p were significantly down-regulated by high-throughput sequencing analysis, which also indicated that miRNAs may be involved in the occurrence of the disease [47].

**Figure 2:**
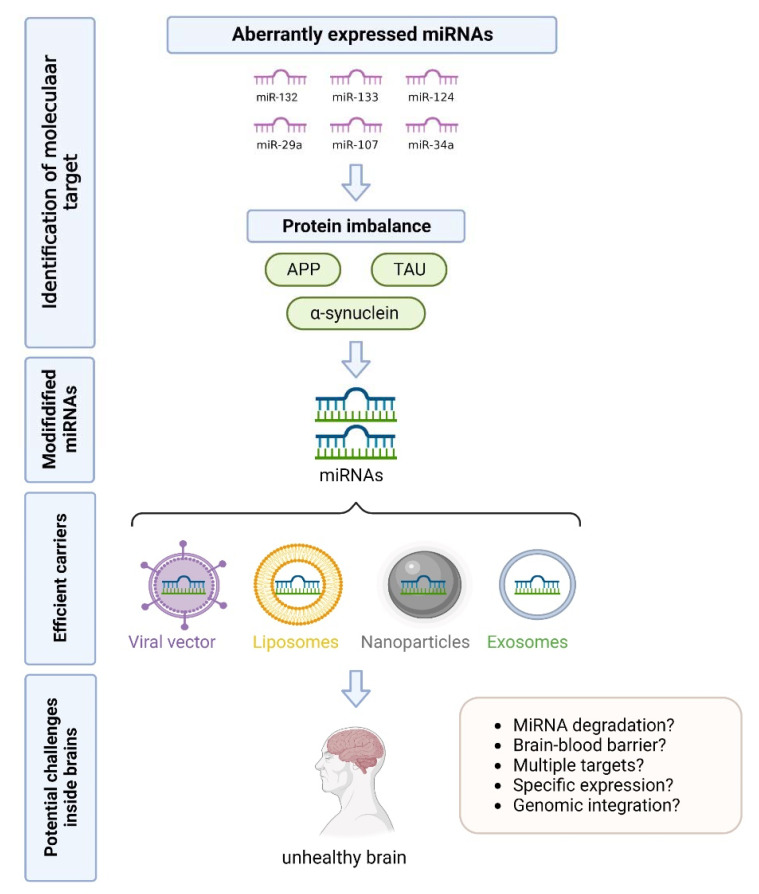
Possible challenges of miRNA-based diagnostics in neurodegenerative diseases.

As more efforts have been made in the roles of miRNAs in different diseases recently, miRNAs have stood out as biomarkers for the early diagnosis of various diseases, especially neurodegenerative diseases [49,50,51]. The study showed that the level of miR-133b was significantly down-regulated in AD patients and Aβ_25-35_-treated SH-SY5Y cells, suggesting that miR-133 could be used for early diagnosis of AD and may also exert neuroprotective effects on AD [52]. Thus, miRNAs are seen as useful tools for the early diagnosis of neurodegenerative diseases. However, the application has been limited due to a series of challenges including the detection methods and effective carriers [53,54,55] (Figure 2). In this review, we describe the dysregulation of miRNA in common neurodegenerative diseases such as AD and PD and present the application of miRNA-based methods in the early diagnosis of these diseases.

## 2. Roles of miRNAs in Neurodegenerative Diseases

**Figure 3:**
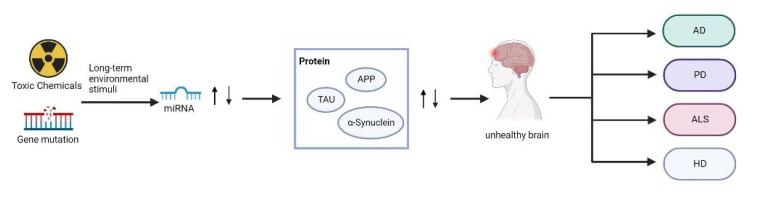
Schematic diagram of the role of miRNA in neurodegenerative diseases.

There are many types of neurodegenerative diseases, and the most common ones are AD, PD, ALS and HD. AD is a neuron-centered disease generally characterized by Aβ and tau phosphorylation [56]. PD is generally characterized by progressive deterioration of motor function due to loss of nigrostriatal dopaminergic neurons with muscle rigidity, bradykinesia and resting tremor [57]. ALS is a fatal onset disease characterized by selective loss of upper and lower motor neurons [58]. HD is a predominantly genetic disease, for which there is no drug cure and it is ultimately fatal [59]. Although their underlying mechanisms remain elusive, many studies have revealed that a series of miRNAs are involved in the development of these diseases [60]. MiRNA regulation happens prior to neurological damage, which emphasizes the significance of miRNA alterations in the disease development. Upregulation/downregulation of miRNA expression leads to the alteration of the protein expressed by the corresponding pathogenic gene, which ultimately results in occurrence and development of neurodegenerative diseases (Figure 3). Therefore, abnormalities of miRNAs have been extensively studied in neurodegenerative diseases, as listed in Table 1.

### 2.1. miRNA in AD

**Figure 4:**
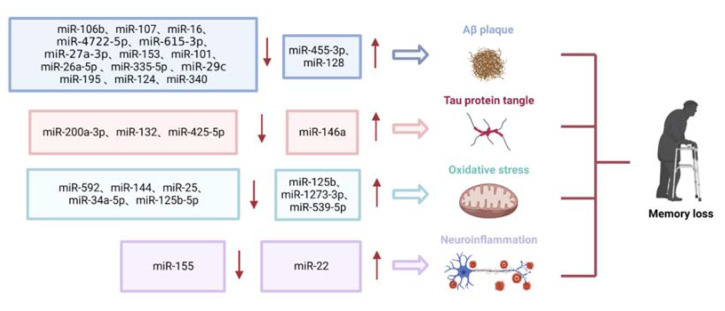
MiRNAs abnormally expressed miRNAs in AD.

AD is the most common neurodegenerative disease that often occurs in people over the age of 65 and affects cognition, memory, language and behavior. It affects around 40 to 50 million people worldwide, but the number of cases is expected to triple by 2050 due to population growth and aging [5]. Although many efforts have been made in the past few decades, the complex pathogenesis of the disease remains unclarified, which limits the development of both diagnosis and treatment methods [100,101,102,103]. The potential of miRNA as biomarkers for early diagnosis of AD has attracted much attention as more and more miRNAs have been found altered in various processes implicated in AD. AD is characterized by two landmarks, the overproduction of Aβ and hyperphosphorylation of tau protein (Figure 4). Moreover, both oxidative stress and neuroinflammation have been reported to contribute to the development of AD [104]. Quite a lot of miRNAs has been reported to be implicated in these processes during AD development. MiR-132, known as “NeurimmiR” due to its involvement in numerous neurophysiological and pathological processes, was identified to be involved in Aβ and tau pathology [105]. Another study showed a few miRNAs such as miR-592, miR-125b and miR-144 were dysregulated, which were associated with AD by regulating oxidative stress [106]. Similarly, a number of miRNAs including miR-155 and miR-146a were investigated and are believed to contribute to the process of neuroinflammation in AD [107].

#### 2.1.1. Role of miRNAs in Aβ Deposition

In AD, the dysregulation of the Aβ level leads to the appearance of senile plaques which contain Aβ depositions. Aβ is a complex biological molecule which interacts with many types of receptors and/or forms insoluble assemblies [69,108]. Aβ is generated by sequential cleavages of amyloid precursor protein (APP) by beta-site APP cleaving enzyme 1 (BACE1) and γ-secretases [109,110]. Its non-physiological depositions alternate with the normal neuronal conditions, impairing synaptic activity and inducing neuritis as well as triggering neurodegeneration. Thus, the role of Aβ deposition in AD has been extensively studied, including the miRNAs involved in this process. For instance, blood samples were collected from 33 AD patients and 33 healthy controls for experiments, and the mRNA expression levels of miR-4722-5p and miR-615-3p were up-regulated in AD [64]. Another study collected serum and cerebrospinal fluid (CSF) samples from 66 AD patients, and the expression levels of miR-27a-3p and NEAT1 in serum and CSF were measured by real-time quantitative PCR experiments. It was concluded that decreased miR-27a-3p levels and increased NEAT1 levels lead to Aβ deposition [63]. To clarify the underlying pathogenesis, both SH-SY5Y cells and rats were treated with amyloid protein and then miR-27a-3p [63]. Results suggest that amyloid protein triggered upregulated NETA1 and downregulated miR-27a-3p and that these effects were improved by miR-27a-3p compensation [63]. The role of NETA1 was further clarified by another two groups and confirmed that NETA1 could sponge-bind miR-107 and miR-24, promote Aβ deposition, and aggravate Aβ-induced neuronal damage [62,111]. By investigating the expression levels of miR-106b in AD patients, experimental studies showed that miR-106b was significantly downregulated in AD [61]. Down-regulation of miR-106b led to an increase in Aβ levels, which might be due to increased expression of BACE1, thereby driving the shift of APP to the Aβ hydrolysis pathway and promoting Aβ deposition [110]. In clinical and mouse model studies, miR-106b expression can be regulated by simvastatin to improve the symptoms of AD [112]. Reduced expression of miR-107 in early AD patients might enable Aβ deposition through regulation of BACE1 [113,114]. In a mouse model of AD, some drugs improved memory loss and reduced Aβ deposition in mice [115]. MiR-29c [65], miR-195 [66], and miR-124 [68] were demonstrated to inhibit the expression of BACE1 by binding with the 3 ‘-UTRs of BACE1. As the levels of these miRNAs decreased in AD, the levels of Aβ increased. The down-regulated expression of miR-16 [69,116], miR-153 [70] and miR-101 [117], which all bind to the 3′-UTRs of *APP*, resulted in an increase in the transcription and protein expression of *APP* and a further increase in the production of Aβ. Another study showed that the expression profile of miR-455-3p was significantly upregulated in AD patients compared to the healthy group [73]. In transgenic AD mice, the expression of miR-26a-5p was reduced, which could be regulated by DYRK1A and overexpression of miR-26a-5p was able to inhibit Aβ deposition [71]. Similarly, in transgenic mice and SH-SY5Y cells, overexpression of miR-335-5p significantly decreased protein levels of Aβ in cells and reduced apoptosis, while inhibition of miR-335-5p produced the opposite result. Furthermore, overexpression of miR-335-5p significantly improved cognitive performance in transgenic mice [67]. MiR-340 was downregulated in AD mice and reduced Aβ accumulation by targeting BACE1 [72]. MiR-128 was upregulated in the cerebral cortex of AD mice and knockout miR-128 suppressed symptoms and reduced Aβ production in AD mice [74]. Therefore, a considerable number of miRNAs play roles in Aβ deposition.

#### 2.1.2. Role of miRNAs in Tau Phosphorylation

Elevated phosphorylation and aggregation of tau protein are widely considered pathological hallmarks of AD [118]. The microtubule-associated tau protein contributes to the stability of axonal microtubules in the brain and is involved in the regulation of axon outgrowth and axonal transport. The binding of tau to microtubules is regulated by post-translational modifications, mostly phosphorylation, which also controls various other less characterized functions of tau [119]. Moreover, tau protein is an important component of neurofibrillary tangles, affecting mitochondrial respiration and synaptic information transmission in neurons [120]. However, the underlying mechanisms remain elusive, which limits the development of effective diagnosing and treatment methods in terms of tau phosphorylation. MiRNAs stand out as potential biomarkers contributing to clarifying the pathogenesis. In animal and cellular models of AD, the expression of miR-200a-3p was suppressed, and miR-200a-3p treatment inhibited apoptosis, inactivated Bax/caspase-3 axis and phosphorylated tau protein [75]. Mechanistically, these effects were mediated by regulating the transport of BACE1 and PRKACB [45]. In detail, the neuroprotective effect of miR-200a-3p was achieved through inhibition of BACE1 expression and subsequent inhibition of Aβ production and reduction of PKA expression and tau phosphorylation [75]. Quite a lot of studies have confirmed downregulation of miR-132 in AD and proposed that miR-132 is involved in AD by controlling apoptosis and tau phosphorylation [76,121,122,123]. Meanwhile, miR-132 expression was shown to be reduced in AD-derived plasma exosomes [76]. Moreover, it was shown that over-expressed miR-425-5p induced apoptosis and promoted tau phosphorylation by targeting the HSPB8 fraction in AD [77]. MiR-146a was also upregulated in AD, and miR-146a adjustment was shown to improve cognitive impairment and alleviate the entire pathological processes, including tau phosphorylation, in *APP/PS1* transgenic mice, a mouse model of AD [78]. Collectively, a series of miRNAs are tightly associated with tau phosphorylation.

#### 2.1.3. Role of miRNAs in Oxidative Stress

Oxidative stress could activate microglia and astrocytes, leading to Ca^2+^ influx and mitochondrial damage in synapses, followed by AD [124]. Oxidative stress is caused by an imbalanced redox state, including overproduction of reactive oxygen species (ROS) or dysfunction of the antioxidant system [125]. The brain is one of the organs particularly vulnerable to ROS because of its high oxygen demand and abundance of peroxidizable fat cells [125]. Physiological changes in these cells may lead to a variety of pathological conditions and human diseases, especially AD [126]. According to numerous studies, oxidative stress has been considered important for the development of AD because it can cause chronic inflammation in the early stages of neurodegeneration, leading to mitochondrial dysfunction, oxidative damage to nucleic acids, changes in gene expression, and abnormal modification of lipids and proteins [104,125,126,127]. A group of miRNAs have been proposed to contribute to these processes. It was indicated that miR-125b induced oxidative stress by inducing Aβ peptide production and sphingosine kinase 1 suppression in an in vitro model of AD [83]. Other studies showed miR-592 and miR-144 modulated oxidative stress by targeting nuclear factor erythroid 2-related factor 2 (Nrf2) in primary astrocytes and SH-SY5Y cells, respectively [79,80]. Similarly, miR-25 affected a form of Aβ-induced oxidative stress by downregulating Nrf2, leading to apoptosis induction [81]. In AD models, overexpression of miR-1273g-3p and miR-539-5p induced oxidative stress, ultimately leading to Aβ production [84,85]. MiR-34a-5p and miR-125b-5p reduced oxidative stress by targeting BACE1, inhibited Aβ-induced neurotoxicity, and provided new targets for AD [82].

#### 2.1.4. Role of miRNAs in Neuroinflammation

Neuroinflammation is also an important factor in the pathogenesis of AD [128]. Neuroinflammation is generally defined as an inflammatory response in the CNS that is caused by trauma, ischemia, infection and other pathological injuries such as toxin accumulation [129]. According to research reports, AD is associated with neuroinflammatory responses, including enhanced astrocyte reactivity, microglia activity, and increased chemokine and inflammatory cytokine loads, which are together believed to promote neurodegeneration [130]. Neuroinflammation is considered to be an important driver in AD, which is generally a chronic process that does not resolve on its own and is associated with the blood-brain barrier and multiple pro-inflammatory factors. There have also been many studies suggesting that AD was closely related to immune mechanisms, which were briefly described here [131,132,133]. In detail, elevated levels of proteins associated with AD pathology and disease severity in AD were positively correlated because these proteins could stimulate receptors on astrocytes and microglia to trigger immune responses, which led to the release of inflammatory mediators [134,135,136]. Another study showed that miR-155 activated astrocytes and led to the production of several pro-inflammatory cytokines (IL-1β, IL-6, and TNF-α) [137]. Activation of inflammasomes in the context of neuroinflammation ultimately led to focal chain cell death by regulating secretion of pro-inflammatory cytokines and cleavage of the N-terminal end of gasdermin D (GSDMD) [138,139]. It was shown that miR-22 expression was reduced in AD patients, and complementation of miR-22 in *APP/PS1* mouse model was able to significantly improve memory and behavior and inhibit the expression of pro-inflammatory cytokines such as IL-1β and IL-1 by suppressing GSDMD [86]. Based on these findings, miRNAs could be considered to play an early diagnostic role in the control of neuroinflammation. Thus, miRNAs could be used as an option for early diagnosis of neurodegenerative diseases.

### 2.2. miRNA in PD

**Figure 5:**
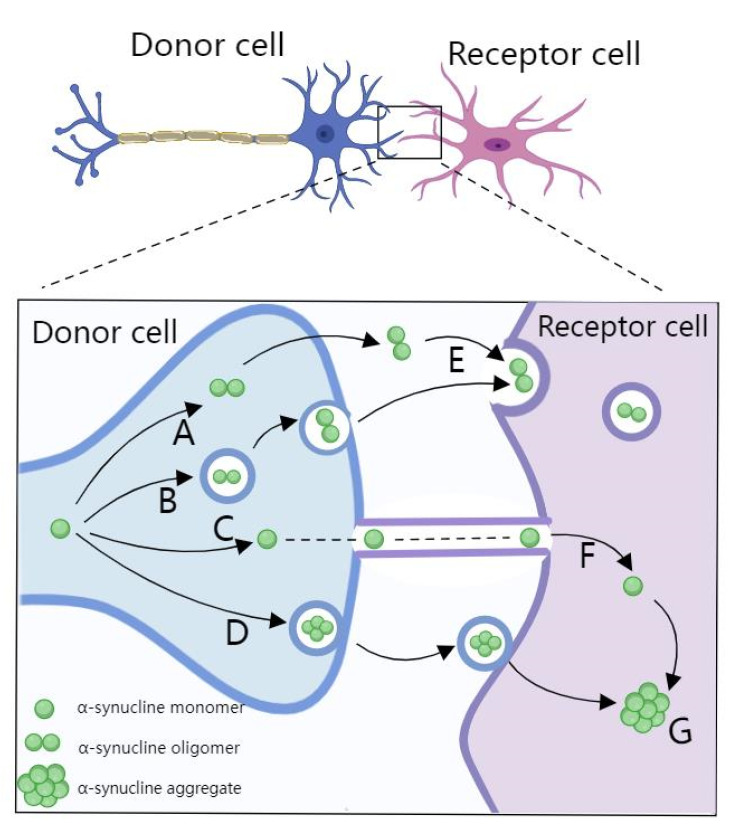
Proposed mechanisms of intercellular α-synuclein transmission. α-synuclein is transferred from donor cells into the extracellular space, as naked protein or in vesicles such as exosomes. They then act as seeds for protein aggregation in the recipient cells. Release mechanisms include the non-vesicular release of free α-synuclein (A), exocytosis (B), transport via tunnelling nanotubules (C), and exosomal transport (D). Uptake mechanisms include endocytosis (E), exosome uptake (F), leading to α-synuclein aggregation formation (G).

PD is the second most common neurodegenerative disease in neurology after AD. The dopaminergic neurons of the midbrain nigrostriatal gradually lose their function and accumulate to a certain extent before the onset of the disease, manifesting as movement disorders and even developing into dementia [140]. It displays as the degeneration and death of dopamine neurons in the brain and causes dementia and mental illness as the symptoms spread to other areas of the brain. Its pathological feature is the formation of a Louis body, which is mainly formed by the aggregation of α-synuclein [141]. Proposed mechanisms of intercellular α-synuclein transmission are shown in [142] Figure 5. A series of miRNAs have been proved to be involved in the pathological processes of PD, such as overexpression of α-synuclein and LRRK2 dysregulation [48]. For instance, Briggs and co-workers showed that miR-744 and miR-532-5p were downregulated, while miR-132, miR-92a, miR-27a and miR-148a were upregulated in brain samples of PD patients [143]. MiRNA can regulate the pathological process of PD through the post-transcriptional expression of α-synuclein and LRRK2, which has become a new tool for the early diagnosis of PD.

Mutations in the α-synuclein gene, which encodes the α-synuclein protein, are known to be one of the main hallmarks of PD [144,145,146]. α-Synuclein are located at the synaptic terminal and widely exist in the adult brain, especially in the neocortex and hippocampus [147]. Overproduced α-synuclein were aggregated to form Lewy bodies, leading to the death of dopaminergic neurons, which in turn triggers PD [148]. Therefore, reducing the expression of α-synuclein through pharmacological intervention might alleviate PD symptoms. A large number of studies have shown that many miRNAs could affect the expression of α-synuclein, some of which have been reported in PD patients [149,150,151]. The most significant miRNAs in α-synuclein expression were probably miR-7 and miR-153 [87]. These two miRNAs reduced α-synuclein levels in PD mice through different pathways, with miR-7 inhibiting its translation and miR-153 degrading mRNA [87,152]. These suggested that they might have a neuroprotective effect in PD patients. In PD patients, miR-7 expression was significantly reduced [153]. It was shown that overexpression of miR-153 and miR-7 in human embryonic kidney cell lines HEK293 cells and cortical neurons led to a significant reduction in α-synuclein mRNA and protein expression levels, while miR-7 knockdown induced overexpression of α-synuclein protein levels [87,153]. Other miRNAs identified as regulators of α-synuclein expression include miR-30b, miR-34b/c, miR-214 and miR-433 [154,155,156].

LRRK2 is a member of the leucine-rich repeat protein kinase family, involved in early neurodevelopmental processes, and its acquired mutations cause familial and sporadic PD [157]. LRRK2 interacts with the miRNA pathway to regulate protein synthesis. LRRK2 mutations result in dopaminergic neuronal degeneration and apoptosis via enhancing LRRK2 kinase activity [158]. LRRK2 mutation could also reduce the expression of miRNAs, because LRRK2 mutation affected the two components argonaute-1 and argonaute-2 in RNA-induced silencing complex, which regulate miRNA functions [159]. MiR-205 was down-regulated in the brain tissue of PD patients, and three transcription factors of the LRRK2 gene were inhibited, resulting in a negative correlation between the expression of LRRK2 protein and the expression of miR-205 [88]. In HEK293T cells, overexpression of miR-205 was involved in inhibiting the expression of LRRK2 protein, whereas inhibiting overexpression of miR-205 enhanced the expression of LRRK2 protein [88]. Overexpression of miR-599 inhibited LRRK2 expression, and downregulation of miR-599 protected SH-SY5Y cells from 1-methyl-4-phenyl-1,2,3,6-tetrahydropyridine induced apoptosis [89].

### 2.3. miRNA in ALS

ALS is a fatal neurodegenerative disease with a mean incidence of 1.8/100,000 and a mean prevalence of 3.40/100,000 in North America [160]. It is characterized by progressive loss of upper and lower motor neurons in the spinal cord, cerebral cortex, and brainstem, resulting in muscle weakness and atrophy, and ultimately paralysis [161]. The pathogenic mechanisms of ALS are poorly understood, although a percentage of patients have familial disease or mutations in genes that are closely related to neuronal function [162,163]. The expression profile of miRNAs stands out as a novel direction for the diagnosis and treatment of the disease [164]. To prove that altered expression of miRNAs is an important factor in ALS disease progression, a recent comprehensive analysis revealed that at least 40 miRNAs were differentially expressed in the muscle tissue of ALS subjects [165].

**Figure 6:**
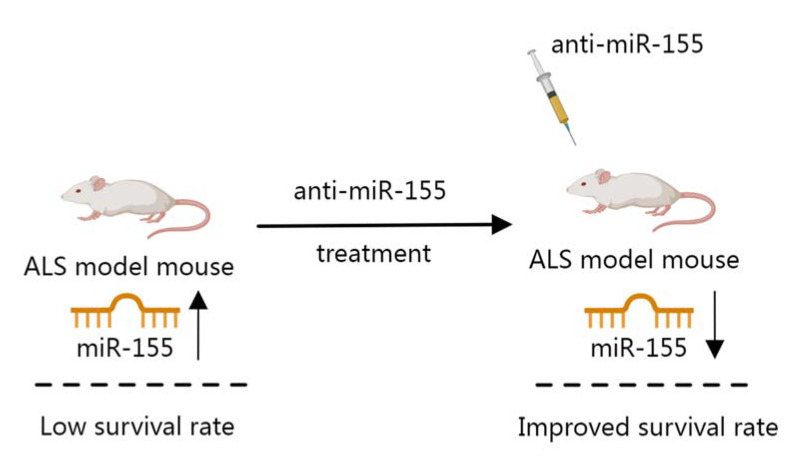
Role of miR-155 in a mouse model of ALS.

With the deepening of the understanding of miRNAs, studies have found that miRNAs are involved in the regulatory process of ALS [166,167]. In ALS mice, miR-206 was found to be abundantly produced, and its upregulation was consistent with the resulting disorder. It was verified by relevant experiments that the lack of expression of miR-206 can slow down the pathological process of ALS and prolong its lifespan [90]. In ALS, another widely studied miRNA is miR-155. The increase of miR-155 in the brain was a detrimental factor for ALS, as the survival rate of rats was increased when miR-155 expression in ALS model mouse brain was inhibited [91] (Figure 6). This suggested that miR-155 has the potential to be a target for the diagnosis of ALS. In the spinal cord of ALS patients, down-regulation of miR-9 and miR-105 targeted the 3’-UTRs of the three intermediate filaments of INA, NEFL and PRPH to regulate gene expression [92]. Therefore, downregulation of these two miRNAs may lead to an imbalance of intermediate filaments, which in turn slows the progression of ALS. Therefore, miRNAs may be a potential strategy for early diagnosis and treatment of ALS.

### 2.4. miRNA in HD

**Figure 7:**
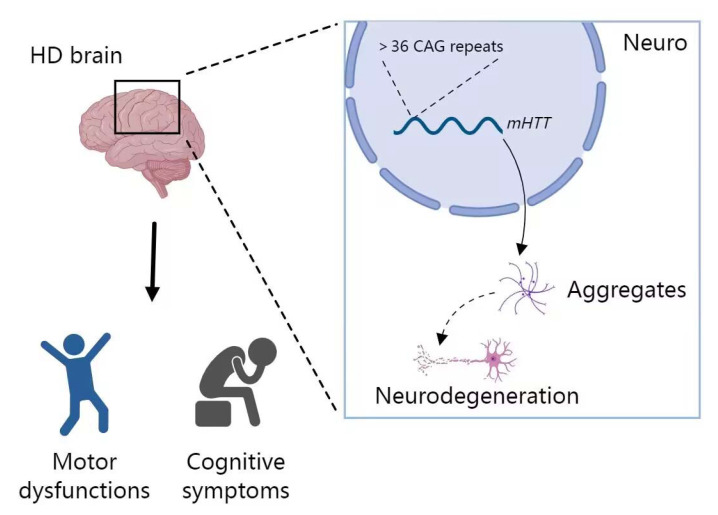
Schematic representation of HD pathogenesis.

HD, as one of the common neurodegenerative diseases, is a genetic and relatively rare disease that is characterized by progressive motor dysfunction, neurocognitive degeneration and brain atrophy [96]. HD is a monogenic disease and the causative gene is *Huntingtin* (*HTT*). Patients carrying mutant *HTT* (*mHTT*) with more than 36 CAG repeats in the exon 1 region of *HTT* will gradually develop HD symptoms [168,169,170,171,172,173,174] (Figure 7). The typical characteristics of HD neuropathology include intranuclear inclusions, nuclear aggregates and neuropil aggregates. The cause of death usually is suicide, as HD patients are unable to tolerate the painful conditions of the symptoms [175,176]. However, except for the symptomatic treatment for motor and psychiatric symptoms, there is no effective treatment for this disease. In recent years, the role of miRNA imbalance in neurological disorders has received increasing attention from researchers in search of new diagnostic approaches and treatment strategies. Several studies have reported the expression profile of miRNAs in HD patients, and altered miRNAs were highly correlated with the regulation of molecular or pathological phenotypes [46,93,170,177,178,179,180,181,182]. Thus, in recent years, aberrant miRNA expression has been reported in HD patients, in vitro experimental models and transgenic HD animal models [93,182,183]. In the near future, dysregulation of miRNAs is likely to be useful for early diagnosis of HD.

An increasing number of studies have shown that miRNAs were dysregulated in HD [57,97,184]. Reed et al. detected a total of 2081 miRNAs by diagnosing HD patients and normal groups, of which miR-520f-3p, miR-135b-3p, miR-4317, miR-3928-5p, miR-8082, miR-140-5p and other miRNAs were expressed at significantly higher levels in HD patients [93]. The expression of two miRNAs, miR-124a and miR-132, was found to be decreased in transgenic HD mice, which was attributed to the abnormal REST leading to increased levels of the target mRNAs of these two miRNAs, further leading to abnormal expression of miR-124a and miR-132 [59]. The expression level of miR-9 was found to be reduced in the cortex of HD patients compared to the normal group, which was also due to the abnormal REST [95]. These studies suggest that miRNAs are extensively involved in the pathogenesis of HD by regulating the target gene *REST*. Due to the limited manipulation of miRNA alterations in HD patients, miRNA studies have been extensively investigated and validated in different animal models. The study reported that miR-128a was downregulated in transgenic HD monkeys, and also confirmed that miR-128a was also downregulated in the brains of pre-symptomatic and post-symptomatic HD patients, suggesting that transgenic HD monkeys and HD patients may exhibit some similar profiles of miRNAs [96]. Similarly, in transgenic mouse studies, miR-34a, miR-124 and miR-132 were suppressed in expression in mouse models, which is similar to HD patients [97,99]. This further suggests that HD patients and animals exhibit some similar miRNA profiles, and these animal models could be used to investigate the possibility of miRNAs as potential diagnostic targets for HD. Indeed, treatment with miR-132 in HD transgenic mice was effective, with some improvement in their behavioral symptoms as well as a delay in the disease [98]. In addition, miR-196a improved the molecular, neuropathological and behavioral phenotypes of HD transgenic mice by enhancing the neuronal cytoskeleton [94]. These further suggest that miRNAs abnormalities have the potential to be used as an early diagnosis of HD.

## 3. MiRNA-Based Bioanalysis in the Diagnosis of Neurodegenerative Diseases

**Figure 8:**
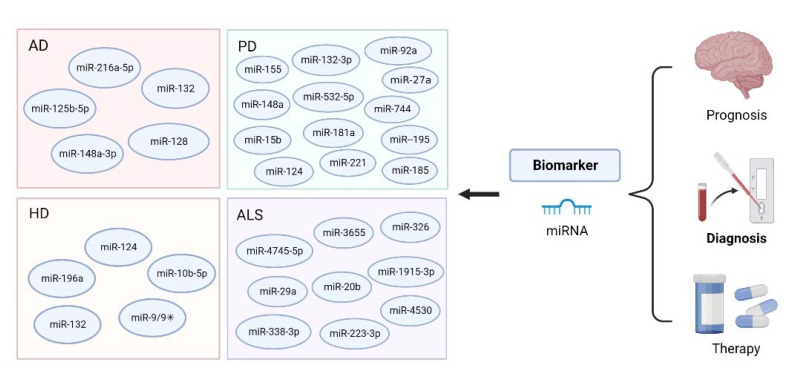
Potential miRNAs for the early diagnosis of neurodegenerative diseases.

Degeneration and dysfunction of neurons are the main features of neurodegenerative diseases, so whether it is possible to reduce the occurrence of the diseases through reliable diagnostic tools that can detect features such as neuronal degeneration in the early stages of the diseases matters. Biomarkers are one of the major tools for the early diagnosis of neurodegenerative diseases and display a series of advantages, such as high sensitivity and specificity, non-invasiveness, ease of handling and high clinical value. There were many biomarkers, such as miRNAs, saliva, piwi-interacting RNAs, neuroimaging and plasma [185,186,187]. When expressed in humans, they not only aid in the identification and analysis of pathological processes, but can also be used to assess different stages of diseases, especially in the early stages [188]. Among the biomarkers, miRNAs stand out in the early diagnosis of neurodegenerative diseases, since these diseases lack non-invasive and effective diagnosis methods and miRNAs are widely present in two extracellular fluids, CSF and blood. Early diagnosis of neurodegenerative diseases is of great significance in reducing their occurrence by early intervention. Conclusively, considering that neurological damage is irreversible and a large number of miRNAs have been proved to be altered in the early stages of neurodegenerative diseases, early diagnosis of neurodegenerative diseases via miRNAs makes a big difference [189] (Figure 8).

### 3.1. miRNA-Based Bioanalysis in AD

**Figure 9:**
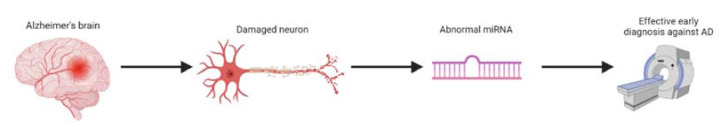
Potential of miRNAs as biomarkers for the early diagnosis of AD.

The expression of many miRNAs has been proved to be altered in AD [190]. Quite a few studies found a number of miRNAs such as miR-125b-5p and miR-148a-3p were down-regulated in the blood of AD patients and emphasized their advantages as diagnostic biomarkers of AD [82,191]. Diana et al. reported measurement of miR-132-3p and miR-212-3p in neurally derived plasma exosomes showed good sensitivity and specificity for diagnosing AD [76]. Another group studied 117 AD patients and showed that the expression of miR-128 in the serum of AD patients was significantly up-regulated, higher than that of the normal group [192]. These demonstrated the great potential of miRNAs in body fluids as candidate diagnostic biomarkers of AD. To further clarify the underlying mechanisms of miRNAs as early-stage diagnostic biomarkers, accumulating studies investigated their signaling pathways with animal models and/or cells of AD. A recent study showed that the level of miR-132 was decreased in AD rats compared with normal rats, resulting in decreased learning ability and increased apoptosis rate in AD rats. These effects were alleviated when an appropriate amount of miR-132 was delivered to AD rats [123]. In another group of AD mice, the expression of miR-216a-5p was down-regulated and AD-related symptoms such as Aβ deposition and neuroinflammation were significantly improved when these mice were treated with miR-216a-5p [193]. Furthermore, in neural stem cells, miR-132 performed as a negative regulator for cell self-renewal and neuronal differentiation but promoted glial cell differentiation and neurite outgrowth [194,195], which are all critical processes supporting brain function. Collectively, miRNA could be potential biomarkers for the early diagnosis of AD as shown in Figure 9.

### 3.2. miRNA-Based Bioanalysis in PD

The abnormal expression of miRNAs has been reported in PD [196]. Briggs et al. found upregulated expression of miR-132, miR-92a, miR-27a, and miR-148a, while miR-744 and miR-532-5p were found to be downregulated, in brain samples of PD patients [143]. Ding et al. reported changes in the expression of five miRNAs in PD serum samples, with miR-15b, miR-181a, miR-185, and miR-221 in downregulation and miR-195 in upregulation [197]. In PD patients and cell models, upregulation of miR-132-3p was confirmed by RNA immunoprecipitation and dual-luciferase reporter gene assays [198]. These studies indicate miRNAs are promising biomarkers for the early diagnosis of PD. To further provide evidence for the method, many investigations on the detailed pathology were carried out with animal models and cell models of PD. Wen et al. demonstrated that overexpression of miR-185 inhibited dopaminergic autophagy and apoptosis of neurons by regulating AMPK/mTOR signaling in PD [199]. MiR-155 plays a central role in the inflammatory response to α-synuclein and α-synuclein-associated neurodegeneration in microglia in the brain [200].

### 3.3. miRNA-Based Bioanalysis in ALS

A group of miRNAs have been reported as prognostic biomarkers for ALS [201]. After an investigation of 252 ALS patients, it was concluded that circulating miR-181 was a prognostic biomarker for the disease [202]. Another study confirmed significant changes of three miRNAs (miR-338-3p, miR-223-3p and miR-326) in the blood samples of ALS patients [203]. It was also shown that four overexpressed miRNAs (miR-1915-3p, miR-3665, miR-4530 and miR-4745-5p) were detected in ALS patients [204]. In addition to clinical results, similar phenomena were found in animal and cell models of ALS [90,205]. Nolan’s team found that miR-29a was highly upregulated in *G93A-SOD1* mice with ALS and suggested that miR-29a might be a potential biomarker of disease progression [205]. MiR-206, a skeletal muscle-specific miRNA, was found to be influenced in *SOD1* transgenic mice, and dysregulation of miR-206 aggravated the progression of ALS [90]. Meanwhile, miRNAs participated in regulating gene expression of the physiological processes of many cells, including cell death [206,207]. Moreover, Zhou and colleagues found that miR-124 is associated with ALS [208]. These findings reveal that miRNA expression changes can be detected before the disease features of ALS are fully manifested, thus allowing for a preliminary determination of ALS.

### 3.4. miRNA-Based Bioanalysis in HD

In HD, miRNA abnormalities can also be used for early diagnosis. Multiple studies investigated the expression profiles of miRNAs in clinical samples of HD patients, and the altered miRNAs were highly correlated with the regulation of molecular or pathological phenotypes in the disease [46,95,182]. The expression of a series of miRNAs such as miR-9/9*, miR-124, and miR-132 were inhibited in both human HD patients and transgenic mouse models [95,99,209]. Treatment with miR-132 in R6/2 HD transgenic mice showed improvement in behavioral symptoms and a delay in disease progression, suggesting that miR-132 could be used not only in early diagnosis, but also for its therapeutic potential [98]. Similarly, miR-196a administration improved molecular, neuropathological, and behavioral phenotypes by enhancing the neuronal cytoskeleton in HD transgenic mice [210,211]. Elevated miR-196a expression levels were observed in the brains of both HD animals and HD patients [182], suggesting miR-196a could be used in early diagnosis of HD. Hoss et al. confirmed that miR-10b-5p was significantly upregulated in HD relative to healthy controls and negatively correlated with the age of onset of HD. Moreover, miR-10b-5p levels were higher when the age of HD onset was earlier [212,213].

## 4. Conclusions and Future Perspectives

Although many studies have identified miRNAs that are aberrantly expressed in neurodegenerative diseases, the underlying pathological mechanisms remain largely unclear. In this paper, we only briefly describe the abnormalities of miRNAs in neurodegenerative diseases. Therefore, there is a need to deeply explore the therapeutic potential of miRNAs in neurodegenerative diseases to reveal innovative approaches in the diagnosis, prognosis, treatment and prevention of neurodegenerative diseases. As in vitro and in vivo studies have demonstrated the neuroprotective potential of certain miRNAs, this would be an interesting area to explore in future studies aimed at developing therapeutic approaches for neurodegenerative diseases. Efforts to achieve up- or down-regulation of desired miRNAs, as well as the development of effective miRNA delivery technologies, would be a promising approach in this direction. Exploring modern molecular biology techniques, miRNA-mediated gene manipulation could be a potential therapeutic strategy for the treatment of neurodegenerative diseases.

MiRNA expression profiles differ between disease states and normal tissue, which make miRNAs promising diagnostic biomarkers. MiRNAs are secreted by cells and stably exist in all kinds of bodily fluids, such as blood, saliva and CSF. This indicates the acquisition of miRNAs is much easier and less invasive than biopsies, especially biopsies of brain. More importantly, miRNA regulation happens prior to the occurrence of reversible damages of neurodegenerative diseases. Thus, miRNAs display obvious advantages as early-stage diagnostic biomarkers of neurodegenerative diseases including AD, PD and ALS. However, although significant advances have been made for the potential of miRNAs in the early diagnosis of neurodegenerative diseases, there have been far fewer clinical applications. The primary difficulties that need to be overcome are the discovery of the miRNAs playing a crucial role in the pathology of a specific disease type and a specific stage of the disease; the comprehensive understanding of the network that miRNAs regulate in a specific disease; and the development of efficient carriers for specific drug delivery. Therefore, more investigations are required to achieve significant progress in these aspects and push miRNAs towards practical applications.

## Figures and Tables

**Table 1:** MiRNAs involved in pathological changes of neurodegenerative diseases.

Neurodegenerative Diseases	MiRNAs	Models	Expression	Pathway Regulation	References
AD	miR-106b	Sera of AD patients	Downregulated	Aβ deposition	[61]
	miR-107	SH-SY5Y and SK-N-SH cells treated with Aβ_1-42_			[62]
	miR-27a-3p	Sera and CSF of AD patients			[63]
	miR-4722-5p, miR-615-3p	Blood of AD patients and PC12 cells treated with Aβ_25-35_			[64]
	miR-29c	miR-29c transgenic mice			[65]
	miR-195	SAMP8 mice and N2a/WT cells			[66]
	miR-335-5p	*APP/PS1* transgenic mice, *APP/PS1* transgenic cells			[67]
	miR-124	PC12 cells and hippocampal neurons treated with Aβ_1-42_			[68]
	miR-16	Brains of AD patients and PC12 cells treated with Aβ_42_			[69]
	miR-153	*APPswe/PSΔE9* transgenic mice			[70]
	miR-26a-5p	*APPswe/PS1* transgenic mice			[71]
	miR-340	SH-SY5Y/*APPswe* cells			[72]
	miR-455-3p	Sera of AD patients	Upregulated		[73]
	miR-128	*APP/PSA/Tau* transgenic mice and N2a cells			[74]
	miR-200a-3p	*APP/PS1* mice, SAMP8 mice, SAMR1 mice, blood of AD patients	Downregulated	Tau protein phosphorylation	[75]
	miR-132	Brains of AD patients			[76]
	miR-425-5p	Postmortem brains of AD patients, HEK293/tau cells, N2a/APP cells			[77]
	miR-146a	*APP/PS1* transgenic mice	Upregulated		[78]
	miR-592	AD rats established by D-galactose and Aβ_25-35_ injection	Downregulated	Oxidative stress	[79]
	miR-144	SH-SY5Y cells treated with Aβ_1-42_			[80]
	miR-25	AD mice established by Aβ_1-42_			[81]
	miR-34a-5p, miR-125b-5p	Sera of AD patients and N2a cells treated with Aβ_25-35_			[82]
	miR-125b	*APPswe/Δ9* transgenic cells	Upregulated		[83]
	miR-1273g-3p	Plasma and CSF of AD patients			[84]
	miR-539-5p	CSF of AD patients and *APP/PS1* transgenic mice			[85]
	miR-22	Blood of AD patients and *APP/PS1* transgenic mice	Upregulated	Neuroinflammation	[86]
PD	miR-7, miR-153	HEK293 cells transfected with miR-7, miR-153	Downregulated	α-synuclein	[87]
	miR-205	Frontal cortex of PD patients	Upregulated	LRRK2 expression	[88]
	miR-599	SH-SY5Y cells treated with MPP^+^		/	[89]
ALS	miR-206, miR-155	*G93A-SOD1* transgenic mice	Upregulated	/	[90,91]
	miR-9, miR-105	Spinal cord of ALS patients	Downregulated	INA, NEFL, PRPH	[92]
HD	miR-520f-3p, miR-135b-3p, miR-4317, miR-3928-5p, miR-8082, miR-140-5p	CSF of HD patients	Upregulated	/	[93]
	miR-196a	HD transgenic mice		/	[94]
	miR-124a, miR-132	Brains of HD patients	Downregulated	/	[59]
	miR-9	Cortices of HD patients			[95]
	miR-128a	Brains of HD monkeys			[96]
	miR-34a, miR-132	*R6/2* transgenic mice			[97,98]
	miR-124, miR-132	Brains of HD patients and cortex and hippocampus of *R6/2* transgenic mice			[99]

CSF, cerebrospinal fluid; APP, amyloid precursor protein; SAMP8, senescence accelerated mice prone-8; N2a/WT, neuro2a/wild-type; *PS1*, presenilin 1; *APPswe*, swedish APP mutant; SAMR1, senescence accelerated mice resistant-1; *SOD1*, superoxide dismutase 1.

## Data Availability

Not applicable.
